# On the Treatment of Ulcers of the Legs by Strapping

**Published:** 1821-01

**Authors:** A. Copland Hutchison

**Affiliations:** Surgeon to his Royal Highness the Duke of Clarence; to the Westminster General Dispensary; to the Royal Metropolitan Infirmary for Sick Children; Medical Superintendant to the General Penitentiary at Millbank, Westminster, &c.


*On the Treatment of Ulcers of the Legs by Strapping.* By A. Copland Hutchison, Esq. Surgeon to his Royal Highness the Duke of Clarence; to the Westminster General Dispensary; to the Royal Metropolitan Infirmary for Sick Children; Medical Superintendant to the General Penitentiary at Millbank, Westminster, &c.

If Mr. Baynton's plan of treating ulcers of the legs by adhesive straps were as generally pursued in the different London hospitals and dispensaries as it is in the royal naval hospitals on the coast, I am thoroughly convinced that its great superiority over every other mode of treatment would soon become apparent, and be more generally adopted in practice.

The expense of strapping in these institutions cannot, surely, be the cause of its almost total disuse; for, when the more speedy recovery of the patient under this treatment is taken into consideration, and hence his or her discharge from the institution the sooner effected, the argument of expense must necessarily fall to the ground.

In the application of the straps and roller, there has been nothing left for future observation by the ingenious deceased author of the plan; and therefore it were almost useless to repeat, that it ought to be done with great care and attention as to smoothness of application and equality of pressure. But there is one circumstance, which, however trifling it may appear, is yet of some consequence, both as it regards a freedom from pain to the patient, expedition, and an appearance of adroitness in the surgeon, which, I believe, has not been noticed by any writer on the subject. It is the manner of removing the old straps in order to replace them by new ones.

Taking off adhesive straps one after another, by detaching an end, and bringing that end round the circumference of the limb, is a tedious process; and, if the hairs on the sound skin, which have been shaved in the first instance, have again grown, considerable pain and inconvenience will be produced. In order to obviate this as much as possible, it has been my plan to insinuate a probe underneath the straps, and in contact with the skin, on the opposite side of the limb to the seat of the ulcer: the straps are thus easily detached from the subjacent parts; and, by gently raising one end of the probe so introduced, a pair of crooked surgeon's scissars, passed in the direction of the probe, will very readily divide the whole at two or three snips of the instrument. The divided ends of the straps are, as a whole united body, to be then turned off to the right and left, so that the ulcer shall be the last part uncovered, and the new-formed skin surrounding the circumference of the ulcer will thus be left undetached from its subjacent tender adhesions.

The hint I am now offering to the profession may appear to some of so trifling a nature, as hardly to merit being recorded; but it has not been so considered, when seen put in practice, by some physicians of eminence in this town; one of whom was the patient, and at whose suggestion I give it publicity through your Journal.

While on this subject, I deem it incumbent on me to notice thus publicly, with a view to its being remedied in the proper quarter, the very parsimonious allowance of adhesive-plaster that is annually supplied to ships of war: and it is, at the same time, but justice in me to state, that every representation of mine to the heads of the medical department of the navy, which related either to the good of the service generally or to the comfort of individuals, being seamen or marines, has hitherto been invariably most promptly attended to.

On referring to Form 91 of the Naval-Hospital Instructions, I find that but three pounds of adhesive-plaster are annually allowed to a frigate of the first class; while ten pounds of Peruvian bark is the allowance for the same period and class of ship, a tenth part of which is possibly never used; and, on enquiry at a respectable wholesale druggist's, I find sticking-plaster only 1*s.* 5*d.* per pound, whilst the best bark is 12*s.*

Let us now examine what will be the effect of a more liberal or proper supply of this most necessary article. There is not any class of medical men more zealous and humane in performing their professional duty than the surgeons of the British navy; and it will be admitted, I believe, on all hands, that there is not a class of men who have greater calls on both. To such gentlemen, therefore, it must be exceedingly vexatious to be under the necessity of sending to an hospital patients labouring under ulcer, who might as well be cured on-board, if the surgeons were sufficiently supplied with the means; and the effective exertions of such men would be thereby equally preserved to the service as if no disease existed; for, it will be readily admitted, I imagine, by those best acquainted with the treatment of ulcer by strapping and a well-applied bandage, that such patients are, generally speaking, as competent to perform the duties of their office whilst under this treatment, as if they were free from disease. Ships of war would not then be deprived of active hands at a period when they are most required and cannot be replaced, and the country would be spared the expense of an hospital treatment.

Again. Suppose a ship to be called into action with the enemy, and amputation performed on one or more men, how distressing would be the situation of the surgeon, who might almost as well be without instruments—(his head, I was about to say,)—as without adhesive-plaster. Had our gallant little squadron, after the battle of Algiers, not gone immediately to Gibraltar, where they were so kindly received and liberally supplied with this necessary article, among others, by the army medical officers of the garrison, I can hardly conjecture what would have been the result to the unfortunate sufferers on that occasion.

Ulcers form a great majority of the cases that fall under the treatment of naval-hospital surgeons, from the cause above adverted to; but, during the several years that I filled the situation of surgeon to men-of-war, previous to being appointed surgeon to Deal Hospital, I find, on a reference to notes, that I had only sent four cases to hospitals; namely, one case of compound fracture of the thigh, one of fractured cranium, one of dysentery, and one case of gun-shot fracture of the leg. Ulcers occurred in the ships of which I was surgeon, as well as in other ships, but I pursued Baynton's plan of treatment, and procured plaster at my own expense to follow it up with; and this I believe to be the reason why no patients of this description were ever sent to an hospital from the ships of which I had the professional charge.

I should wish it to be understood, however, that where the malignant ulcer, or, as it is sometimes called, hospital gangrene, occurs on-board ships of war, the sooner such patients are removed to an hospital, away from the crew, the better; and it is scarcely necessary for me to add, that these are not cases to be treated by adhesive straps: but, with regard to the particular treatment of malignant ulcer, I shall, I trust, have more to say at some future opportunity.

I beg pardon for this digression. I have only been acquitting myself of a public duty, which I have now no other means of doing but through the medium of the press.

*Spring Garden; 9th Dec.* 1820.

